# Mexico's Health System: More Comprehensive Reform Needed

**DOI:** 10.1371/journal.pmed.1000130

**Published:** 2009-08-18

**Authors:** Jason Lakin

**Affiliations:** Department of Global Health and Population, Harvard School of Public Health, Boston Massachusetts, United States of America

## Abstract

Jason Lakin discusses and critiques a Policy Forum that reviews 25 years of reform to the Mexican health system and argues that more comprehensive reform is needed.

**Linked Policy Forum**

This Perspective discusses the following new study published in *PLoS Medicine*:

Homedes N, Ugalde A (2009) Twenty-Five Years of Convoluted Health Reforms in Mexico. PLoS Med 6(8): e1000124. doi:10.1371/journal.pmed.1000124

Nuria Homedes and Antonio Ugalde discuss 25 years of reform to the Mexican health care system and argue that although costs and accessibility have increased, health inequities, efficiency, productivity, and quality of care have not improved.

## Introduction

The article by Homedes and Ugalde in this week's issue of *PLoS Medicine* is an important addition to the global discussion about Mexico's 2003 health reform, the Seguro Popular (SP) [1]. While this reform has been controversial in Mexico [2], it has been highly praised in international circles. *The Lancet *ran a special series on the reform in 2006, and former U.S. President Bill Clinton lauded SP in a speech last year at the International AIDS Conference [3],[4].

In spite of this high-profile attention, there has been little written about the reform by independent observers in English. *The Lancet* series was largely authored by the reformers themselves, along with close allies. Although a few technical studies have now come out demonstrating some impact of the reform on various outcomes [5],[6], broader analysis of the institutional structure of the reform, and the context for its creation and implementation, are almost completely lacking.

Homedes and Ugalde have finally given us this kind of broader analysis and historical context, and they deserve immense credit for doing so. Their piece covers a large period of time and number of variables. In this Perspective, I parse some of the key issues they raise about Mexico's health system, and point to some areas of disagreement. I conclude with some issues that they do not address.

## Key Issues

Homedes and Ugalde raise several points about the history of Mexico's health reforms. One of the most important is the claim that the federal government has shifted back and forth between centralizing and decentralizing reforms in a way that has impeded capacity building within the health sector. In particular, the government decentralized in the late 1990s, but then SP recentralized key elements of the health system. This allegation is contentious, because the authors of the 2003 reform have argued that SP continues the trend toward decentralization [7].

Homedes and Ugalde are correct, however: The reform explicitly attempts to undo one aspect of the prior decentralization, by redirecting health funds for state health services away from unconditional block grants and toward a centralized, conditional fund. States are forced to create structures that permit the devolution of funds on a conditional basis, to affiliate their citizens in order to get access to central resources, and to provide services on a list set by the federal government. Like most reforms related to centralization/decentralization, not everything moves in the same direction: states have more freedom to contract with the private sector under the new system, which can be seen as increasing decentralization. On balance, however, SP is a centralizing reform.

The second major claim that Homedes and Ugalde make is that the reforms of the last 25 years, including SP, have injected new money into the health system, but have done little to increase quality or efficiency. The precise source of the problem is not identified, but one can tease out a few issues from the article. First, there is low productivity in the public health service. Second, there are excessively high administrative costs. Third, there is a high level of “bureaucratic rigidity” and a low level of state “managerial capacity.”

Few would disagree with these claims, but their precise relationship to the reforms of recent years is not entirely clear. Did the reforms cause these problems, or simply fail to address them? Or are these problems primarily of interest because they have scuttled the implementation of reforms?

Homedes and Ugalde do not provide any clear alternative for dealing with low efficiency or quality in the health sector. Other analysts, however, have suggested that increasing the role of the private sector in the health system could lead to improved efficiency and quality. As it happens, the increasing role of the private sector in Mexico is a third major issue raised by Homedes and Ugalde. Their distaste for increasing the role of private actors in the health sector is clear, but not their rationale. This is unfortunate, since the rationale in favor of private provision has been clearly stated by the reformers: lack of competition in the public sector has led to a lack of incentives to provide quality care at a reasonable price [8]. Do Homedes and Ugalde contest this? If not, how would they propose to fix the problem without introducing private competition?

There is also an empirical question here: to what degree does SP promote private interests in the health sector? While Homedes and Ugalde are correct to identify a trend toward increasing private participation in Mexico's health system, they are less careful in exploring the vision of the private sector's role held by SP. Like decentralization, this is an important and contentious issue. Many Mexicans believe that SP represents a stealth attempt to privatize the Mexican health system [9]. Homedes and Ugalde refer to these “critics” approvingly, and seem to agree.

In my view, the claim that SP is privatizing Mexico's health system is questionable. While the reform clearly allows for more private sector participation in terms of provision, it also bolsters public infrastructure, increases the number of public doctors and health workers, and improves their working conditions (as Homedes and Ugalde note). The government is encouraging both public and private *contracting*, but has not turned its back on the public sector in favor of private provision. In addition, SP represents an infusion of public finance and a reduction in private out-of-pocket expenditure on health [5],[10]. None of this is consistent with a logic of privatization.

## Moving Forward

Homedes and Ugalde acknowledge that SP has had some successes. In general, they believe that the reforms of recent decades (including SP) have increased access to care. They also allow that a decrease in out of pocket costs has occurred. In spite of this, Homedes and Ugalde believe that SP moves Mexico in the wrong direction.

This is a fair point of view, but I am not sure Homedes and Ugalde provide an alternative path to improving the Mexican health system. They suggest that working through social security (IMSS, the Instituto Mexicano del Seguro Social) might have been a better approach, but sidestep the problem of how to increase efficiency in IMSS without taking on the institution's powerful provider union or introducing competition.

A more intriguing suggestion is that the reformers could have simply eliminated user fees in public clinics by infusing more cash to cover them, without creating a new, complex system. Given that SP really does not work like insurance (97% of families do not pay a premium [11], and most states also have not paid their share of the premium [10],[12]), that services are not really guaranteed (as they show), and that there is little evidence of increased utilization but only of reduced out of pocket expenditures [5], it is worth asking whether the complex structure of SP, which incurs substantial transaction costs, is better than simply eliminating user fees, hiring more doctors, and keeping the previous system in place.

The problem with this option is that it fails to address key problems with the existing system. To be sure, the utilization of regressive user fees is one of those problems. But how should the Mexican health system deal with absenteeism and low productivity among medical workers, inability to staff marginalized areas of the country, and insufficient training of medical residents and clinicians to confront the epidemiology of rural and indigenous areas? The system requires a restructuring of labor relations and the professionalization of the union and its constituent health workers.

Homedes and Ugalde are probably right to doubt that private competition alone will fix these problems, but they present no alternatives. For all its flaws, contracting out is at least premised on a theory of how to make services more attentive to patients. That theory may be wrong (there are numerous market failures in health care), but it would have been helpful had Homedes and Ugalde taken it on more directly, offering some politically feasible strategy for improving professional management of Mexico's delivery system.

Other issues that are essential to improving Mexico's health system are tackled neither by SP nor by Homedes and Ugalde. First, in the absence of a major tax reform, and given the failure of states and families to pay their share of premiums, how can Mexico generate sufficient revenues to pay for the level of health service Mexicans deserve? Second, what complementary services, such as transportation, translation, or additional training, are needed to make public services accessible to those who need them most (rural poor, indigenous, etc.)? Third, how should quality and financing issues in the rest of the health system, primarily IMSS, be resolved?

For all of its flaws, Seguro Popular has made an attempt to improve a part of the Mexican health care system. The program deserves critical analysis so that others may learn from it. Homedes and Ugalde have pushed the international discussion of the program forward. They leave us with many unanswered questions. It is to be hoped that continuing debate and dialogue will lead us closer to the answers.

## Abbreviations

SP: Seguro Popular
IMSS: Instituto Mexicano del Seguro Social
